# Effects of continuous tillage on soil fungal and bacterial communities at sandy lined-film paddy in Horqin Sandy Land, China

**DOI:** 10.3389/fmicb.2026.1807818

**Published:** 2026-04-10

**Authors:** Anqi Cong, Jin Zhan, Ning Wang, Tonghui Zhang

**Affiliations:** 1 Inner Mongolia Naiman Agroecosystem National Field Observation and Research Station, Northwest Institute of Eco-Environment and Resources, Chinese Academy of Sciences, Lanzhou, China; 2 State Key Laboratory of Ecological Safety and Sustainable Development in Arid Lands, Northwest Institute of Eco-Environment and Resources, Chinese Academy of Sciences, Lanzhou, China; 3 College of Resources and Environment, University of Chinese Academy of Sciences, Beijing, China; 4 Gansu Minqin National Field Observation and Research Station on Ecosystem of Desert Grassland, Gansu Desert Control Research Institute, Lanzhou, China; 5 Institute of Crop Sciences, Gansu Academy of Agricultural Sciences, Lanzhou, China

**Keywords:** artificial paddy fields, diversity, entisols, Horqin Sandy Land, soil microbial community

## Abstract

Soil microorganisms play an important role in energy flow and nutrient cycling between soil matrix and plants, but the effect of continuous cultivation on soil bacterial and fungal communities in artificial paddy with lined-film practice remains uncertain. Therefore, we investigated the microbial diversity and community of a soil lined-film with rice grown continuously for a maximum of 27 years. Our results showed that the α-diversity of soil bacterial communities was higher than that of fungi under continuous cultivation. Continuous tillage had a greater effect on the soil bacterial community than on the fungal community, and the fungal community was more stable than the bacterial community. Bacteria were mainly dominated by Actinobacteria, Proteobacteria and Firmicutes, while fungi were dominated by Ascomycota and Basidiomycota. In addition, the fungal Chao 1 index was significantly affected by SOC and TN. Soil pH is an important driver of soil microbial communities under fertilization conditions in sandy artificial paddy field. The results of this study provide empirical support for the sustainable development of lined-film paddy cultivation in sandy farmland ecosystems. They offer a basis for formulating fertilization management strategies that maintain microbial diversity, ensure sustainable land development, and combat desertification.

## 1 Introduction

Soil microorganisms, especially fungi, play an important role in the formation and maintenance of soil structure (Yarzábal Rodríguez et al., 2024). They contribute to the formation of soil microaggregates through the secretion of cementing substances, which in turn affects the water retention capacity and aeration of the soil, thereby promoting healthy crop growth (Hartmann and Six, 2023). In highly disturbed agricultural ecosystems, tillage practices can directly or indirectly influence the biomass and composition of soil microbial communities by altering soil physicochemical conditions (Pires et al., 2017; Zhang et al., 2019). Different tillage methods exert significant impacts on microbial activity and soil nutrient cycling within farmland ecosystems (Zhang D. X. et al., 2022). Agricultural management practices such as tillage and straw incorporation substantially affect the community structure and diversity of soil microorganisms. Extensive research indicates that appropriate agricultural management practices positively influence soil nutrients and microorganisms. For instance, applying organic fertilizers and tillage enhance soil microbial diversity and organic matter content (Xu et al., 2021; Song et al., 2022).

The application of lined-film technology in sandy paddy fields is a management measure designed to eliminate fertilizer loss and water seepage in dune soils, enhance water use efficiency, and control soil erosion. This technology primarily involves laying a water-impermeable layer (plastic film) within the soil layer after leveling mobile and semi-mobile sand dunes. This addresses the drawbacks of sandy soil—water and fertilizer leakage—and the challenges posed by uneven terrain that hinders cultivation. By leveraging the favorable physicochemical properties of wind-blown sandy soil—its loose texture, rapid heat retention, and efficient nutrient conversion—the technique not only achieves high yields but also conserves water and fertilizer (Zhang et al., 2006). By fully leveraging the land resources, solar heat resources, and relatively abundant water resources of semi-arid sandy areas, the biomass of sand dune land has been significantly increased from less than 32.1 to 1,200 kg per mu after rice cultivation (Dou and Sun, 2017). Through irrigation, nitrogen supplementation via organic and chemical fertilizers, and straw return practices, the lined-film paddy technique fundamentally improves sandy dune soil conditions.

Chemical fertilizers and organic fertilizers are the most commonly used fertilization methods for paddy soils. While chemical fertilizers can boost crop yields in the short term, they may lead to soil acidification, nutrient imbalance, and reduced microbial diversity. Organic fertilizers improve soil physicochemical properties by increasing soil organic carbon and readily available nutrients, promoting the proliferation of beneficial microorganisms and thereby enhancing the stability of soil ecosystems (Zhang et al., 2010; Dai et al., 2021). Appropriate nutrient management practices (such as crop straw incorporation, organic fertilizer application, and combined organic-inorganic fertilizer application) directly influence changes in soil organic carbon (SOC) and organic nitrogen (SON) components (Wissing et al., 2011). Studies indicate that in dryland agriculture, straw incorporation significantly increases SOC, easily oxidizable organic carbon (EOC), and microbial biomass carbon (MBC) content (Fan and Wu, 2020). Combined application of organic and inorganic fertilizers primarily increases paddy soil particulate organic carbon (POC), acid-hydrolyzable amino acid nitrogen, acid-hydrolyzable unknown nitrogen, and non-acid-hydrolyzable nitrogen, promoting SOC and SON accumulation (Huang et al., 2009). Fertilization significantly alters paddy soil microbial community structure; long-term exclusive chemical fertilizer application disrupts microaggregate structure and reduces soil microbial activity (Liu et al., 2025). Applying organic fertilizer maintains soil microbial activity and enhances microbial diversity (Jiang et al., 2024). The combined application of organic and inorganic fertilizers optimizes the microbial community structure in red-soil paddy fields, significantly boosting soil microbial activity (Zhang Q. et al., 2022; Wang et al., 2024).

Since the early 1990’s, Naimanqi and Kulunqi in Tongliao City have promoted this technology that combines yield enhancement with desertification control. Through artificial means, gently sloping sand dunes were converted into rice paddies. After its development, this technology was vigorously promoted. However, due to various factors—such as switching to corn cultivation and fruit tree planting (which caused damage to plastic mulch)—the area under rice cultivation has sharply declined. Due to the relatively short duration of technological development and limitations in cultivation areas, research on this farming method remains scarce. Therefore, this experiment, based on the longest existing continuous 27-year cultivation record, investigates the driving factors behind changes in soil physicochemical properties, microbial structural composition, and microbial community variation in plastic-covered paddy fields across semi-arid and desertified regions under different cultivation durations. The findings will provide scientific support for enhancing the quality and efficiency of local plastic-lined paddy fields.

## 2 Materials and methods

### 2.1 Study area

The study area is located in Kulunqi (42°21’-43°14’ N, 121°09’-122°21’ E), at the southern edge of the Horqin Sandy Land in the agro-pastoral transition zone of northern China. It features a temperate semi-arid continental monsoon climate, with annual average evaporation ranging from 1,982 to 2,210 mm, with an annual average temperature of 6.2 °C–6.8 °C, a frost-free period of 143–156 days, accumulated temperature ≥ 10 °C of 3,000 °C–3,200 °C, annual precipitation of 383.7–447.5 mm, and wind speeds ranging from 3.7 to 4.4 ms^–1^. Demarcated by the local Yangxumu River, the northern region predominantly features sandy loam soils, while the southern area exhibits greater soil diversity, including chestnut brown soils, sandy loam, coarse skeletal soils, stony soils, and brown soils (Li et al., 2021). This study was conducted in northern Kulunqi. Following the development of lined-film paddy technique, its adoption expanded significantly in sandy areas, reaching a peak planting area of 30,000 mu (approximately 2,000 hectares) across the region (Bao et al., 2019). Subsequently, planting areas decreased due to crop rotation to maize and other factors. Surveys indicate that the rice planting area in 2023 was approximately 3,000 mu (approximately 200 hectares).

### 2.2 Experimental design and soil sampling

Preliminary surveys of sandy paddy fields were conducted in Elesun Town and Manghan Sumu, Kulunqi in 2022. Currently, long-term cultivation is primarily carried out by individual farmers, with plots mostly distributed around homes and varying in size. Base fertilizer consists mainly of farmyard manure, while top dressing relies predominantly on chemical fertilizers. Plots with comparable cultivation areas and consistent fertilization, routine management, and irrigation practices were selected. Soil samples were collected from sandy paddy fields with varying cultivation durations-1, 5, 10, 15, 20, and 27 years-as well as from adjacent sand dune soil (CK) in November 2023, following rice harvest. Each cultivation duration had 4 replicates. Soil was collected using the five-point method in layers from 0 to 10 cm and 10 to 20 cm. Soil from the same layer was thoroughly mixed and sealed in self-sealing bags. Samples were sieved through a 2 mm mesh to remove visible impurities, fallen leaves, stones, and plant roots. The samples were then divided into two groups: one group was placed on dry ice immediately after collection and transported to the laboratory, where it was stored at −80 °C for high-throughput DNA sequencing analysis. The other group was air-dried naturally for determining soil physicochemical parameters.

### 2.3 Soil physicochemical properties measurement

Soil pH and electrical conductivity (EC) were measured using a HACH portable electrochemical analyzer (HQ30d). Soil organic carbon (SOC) was determined using the potassium dichromate oxidation-spectrophotometric method. Total nitrogen (TN) was analyzed with an elemental analyzer (Costech ECS 4010), while total phosphorus (TP) was measured via digestion-spectrophotometric analysis using the molybdenum-antimony reagent (Institute of Soil Science, Chinese Academy of Sciences [ISSCAS], 1978).

### 2.4 Measurement of soil microbial community

Total microbial genomic DNA was extracted from soil samples using the E.Z.N.A^®^ soil DNA Kit (Omega Bio-tek, Norcross, GA, United States) according to manufacturer’s instructions. The quality and concentration of DNA were determined by 1.0% agarose gel electrophoresis and a NanoDrop2000 spectrophotometer (Thermo Scientific, United States) and kept at −80 °C prior to further use. The hypervariable region V3–V4 of the bacterial 16S rRNA gene were amplified with primer pairs 338F (5′-ACTCCTACGGGAGGCAGCAG-3′) and 806R (5′-GGACTACHVGGGTWTCTAAT-3′) (Liu et al., 2016) by T100 Thermal Cycler PCR thermocycler (BIO-RAD, United States). The fungal 18S rRNA gene were amplified with primer pairs SSU0817F (5′-TTAGCATGGAATAATRRAATAGGA-3′) and 1196R (5′-TCTGGACCTGGTGAGTTTCC-3′). The PCR reaction mixture including 4 μL 5 × Fast Pfu buffer, 2 μL 2.5 mM dNTPs, 0.8 μL each primer (5 μM), 0.4 μL Fast Pfu polymerase, 10 ng of template DNA, and ddH_2_O to a final volume of 20 μL. PCR amplification cycling conditions were as follows: initial denaturation at 95 °C for 3 min, followed by 27 cycles of denaturing at 95 °C for 30 s, annealing at 55 °C for 30 s and extension at 72 °C for 45 s, and single extension at 72 °C for 10 min, and end at 4 °C. The PCR product was extracted from 2% agarose gel and purified using the PCR Clean-Up Kit (YuHua, Shanghai, China) according to manufacturer’s instructions and quantified using Qubit 4.0 (Thermo Fisher Scientific, United States).

Purified amplicons were pooled in equimolar amounts and paired-end sequenced on an Illumina Nextseq2000 platform (Illumina, San Diego, United States) according to the standard protocols by Majorbio Bio-Pharm Technology Co., Ltd., (Shanghai, China).

Bioinformatic analysis of the soil was carried out using the Majorbio Cloud platform.^1^ Based on the OTUs information, rarefaction curves and alpha diversity indices including observed OTUs, Chao1 richness, Shannon index and Good’s coverage were calculated with Mothur v1.30.1 (Schloss et al., 2009). The similarity among the microbial communities in different samples was determined by principal coordinate analysis (PCoA) based on Bray-curtis dissimilarity using Vegan v2.5-3 package. The PERMANOVA test was used to assess the percentage of variation explained by the treatment along with its statistical significance using Vegan v2.5-3 package. The linear discriminant analysis (LDA) effect size (LEfSe) (Segata et al., 2011)^2^ was performed to identify the significantly abundant taxa (phylum to genera) of bacteria among the different groups (LDA score > 2, *P* < 0.05). We performed non-metric multidimensional scaling (NMDS) analysis of soil microbial communities through the vegan package in R (version 3.6.2) using Bray-Curtis. Amos 22.0 was used to conduct SEM.

### 2.5 Statistical analysis

Preliminary data aggregation and processing were conducted using Microsoft Excel 2019 software. Normality tests were performed on all variable data using the S-W test, confirming that all data met the assumption of normal distribution and were suitable for direct statistical analysis. SPSS 25.0 software was employed for one-way analysis of variance (ANOVA) to examine the effects of different rice cropping years on each variable indicator, with *p*-values (*p* < 0.05, *p* < 0.01, *p* < 0.001) used to determine the significance of variation between them. Origin 2026 software was utilized for data visualization. Finally, Pearson correlation coefficient models were applied to evaluate the correlated changes between physicochemical properties and microbial diversity in sandy paddy soils of semi-arid regions.

## 3 Results

### 3.1 Soil physical and chemical properties

Physical and chemical properties of soils differed among tillage years. With increasing cultivation duration, soil pH significantly decreased to 5.7. Meanwhile, soil electrical conductivity markedly increased with rice cultivation duration, with the greatest increase observed in the topsoil layer, reaching 363.59%. This significantly impacted soil organic carbon content. Organic carbon in the topsoil (0–10 cm) increased from 1.24 to 3.72 g⋅kg^–1^, a rise of 200.0%. In the 10–20 cm layer, organic carbon content rose from 1.27 to 3.63 g⋅kg^–1^, an increase of 185.8%. With increasing years of cultivation, soil total nitrogen content significantly increased. In the topsoil (0–10 cm), total nitrogen content rose significantly from 0.18 to 0.47 g⋅kg^–1^, an increase of 161.1%. While in the 10–20 cm layer, it rose from 0.15 to 0.35 g⋅kg^–1^, representing a 133.3% increase. Total phosphorus (TP) content showed a slight increase. Soil carbon-to-nitrogen ratio (C:N), carbon-to-phosphorus ratio (C:P), and nitrogen-to-phosphorus ratio (N:P) all exhibited upward trends (Table 1).

**TABLE 1:** Physico-chemical properties of soils with various tillage years (means ± SE).

Tillage	pH	EC μS⋅cm^–1^	SOC g⋅kg^–1^	TN g⋅kg^–1^	TP g⋅kg^–1^	C:N	C:P	N:P
0–10 cm								
CK	7.02 ± 0.06a	31.17 ± 5.30b	1.24 ± 0.40b	0.18 ± 0.03c	0.14 ± 0.01c	6.56 ± 1.64a	9.63 ± 3.69b	1.36 ± 0.28c
1Y	7.00 ± 0.39a	63.75 ± 7.65b	1.23 ± 0.26b	0.19 ± 0.01c	0.14 ± 0.01bc	6.70 ± 1.82a	8.36 ± 1.62b	1.31 ± 0.09c
5Y	6.37 ± 0.40ab	133.25 ± 15.89a	1.72 ± 0.23b	0.27 ± 0.06bc	0.16 ± 0.01ab	6.56 ± 0.47a	10.43 ± 1.31b	1.65 ± 0.32bc
10Y	6.16 ± 0.26ab	151.53 ± 21.73a	1.53 ± 0.17b	0.27 ± 0.04bc	0.17 ± 0.01a	5.92 ± 0.72a	9.09 ± 1.89b	1.60 ± 0.25bc
15Y	6.03 ± 0.23b	153.98 ± 26.65a	3.56 ± 0.58a	0.38 ± 0.06ab	0.17 ± 0.01a	9.87 ± 1.52a	21.05 ± 3.20a	2.24 ± 0.40abc
20Y	5.70 ± 0.18b	150.70 ± 23.48a	3.43 ± 0.62a	0.42 ± 0.06ab	0.17 ± 0.01a	8.32 ± 1.23a	21.04 ± 4.35a	2.55 ± 0.41ab
27Y	5.91 ± 0.32b	144.50 ± 14.52a	3.72 ± 0.27a	0.47 ± 0.06a	0.16 ± 0.01ab	8.39 ± 1.65a	22.60 ± 1.90a	2.88 ± 0.39a
10–20 cm								
CK	6.98 ± 0.02ab	31.00 ± 5.12c	1.27 ± 0.34b	0.15 ± 0.02c	0.14 ± 0.01a	7.89 ± 1.37a	10.28 ± 3.84a	1.19 ± 0.29b
1Y	7.15 ± 0.26a	67.75 ± 9.23bc	1.42 ± 0.50b	0.17 ± 0.02c	0.14 ± 0.01a	7.82 ± 2.03a	9.68 ± 3.15a	1.17 ± 0.10b
5Y	6.90 ± 0.34ab	159.30 ± 23.55a	2.13 ± 0.30ab	0.26 ± 0.03abc	0.16 ± 0.01a	8.30 ± 0.65a	13.21 ± 1.56a	1.60 ± 0.16ab
10Y	6.70 ± 0.24abc	136.93 ± 26.37a	1.91 ± 0.37b	0.23 ± 0.03bc	0.17 ± 0.01a	8.27 ± 0.48a	11.32 ± 2.46a	1.34 ± 0.22b
15Y	6.57 ± 0.32abc	120.73 ± 5.17ab	3.47 ± 0.66a	0.29 ± 0.04ab	0.17 ± 0.01a	12.36 ± 2.60a	20.70 ± 3.83a	1.74 ± 0.26ab
20Y	6.01 ± 0.14c	145.88 ± 24.47a	3.53 ± 0.72a	0.31 ± 0.06ab	0.16 ± 0.02a	12.52 ± 3.19a	23.68 ± 7.75a	1.88 ± 0.28ab
27Y	6.29 ± 0.04bc	108.55 ± 11.84ab	3.63 ± 0.40a	0.35 ± 0.03a	0.16 ± 0.01a	10.55 ± 1.61a	23.64 ± 4.53a	2.23 ± 0.19a

Different lowercase letters indicate significant difference among various tillage years (*p* < 0.05). pH, soil pH value; EC, soil electrical conductivity; SOC, soil organic carbon content; TN, soil total nitrogen content; TP, soil total phosphorus content; C:N, soil C:N ratio; C:P, soil C:P ratio; N:P, soil N:P ratio; CK, control; Y, year.

### 3.2 Soil microbial α- and β-diversity

**FIGURE 1:**
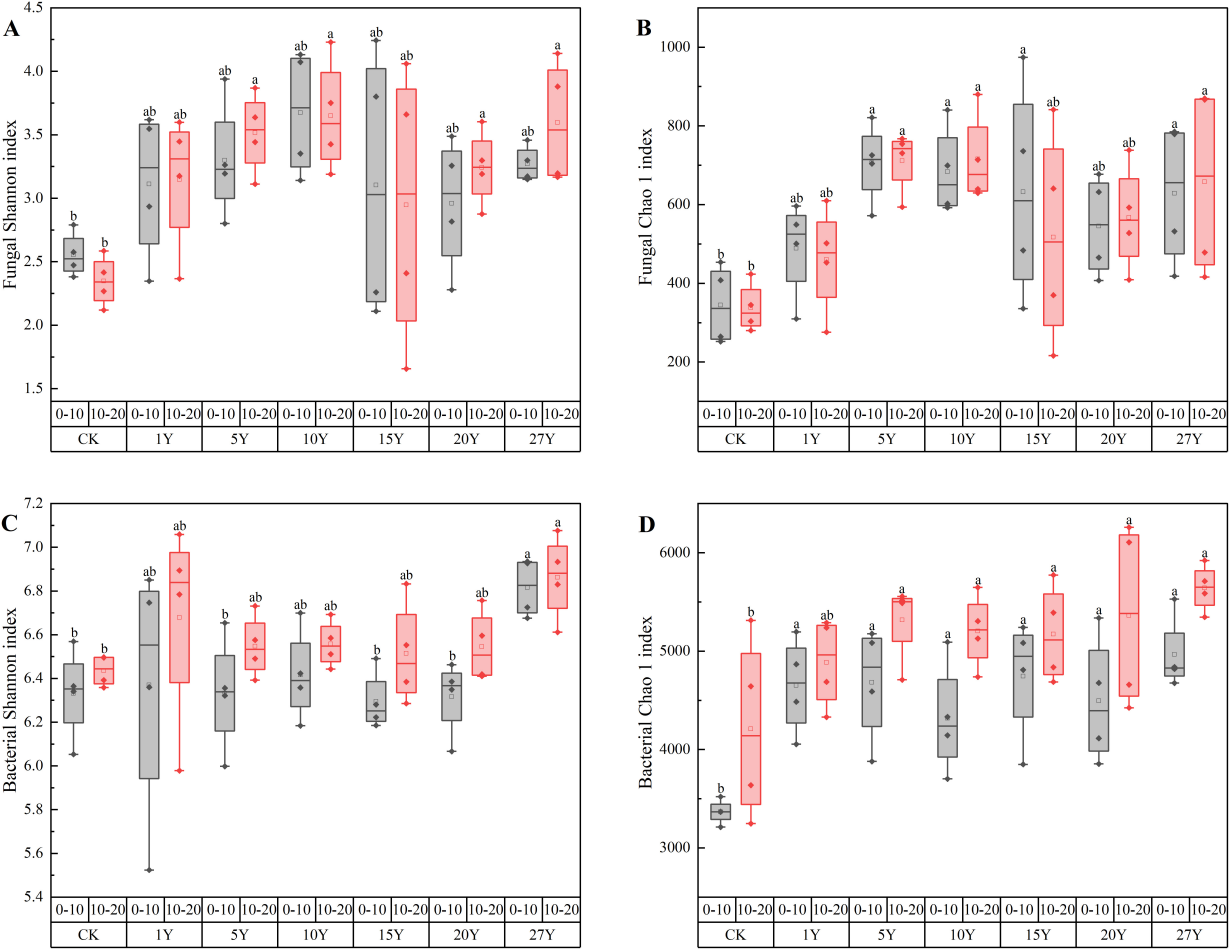
Changes in soil fungal and bacterial Shannon diversity indices and chao1 abundance in rice planted during 0–27 years. Different lowercase letters indicate significant difference among various tillage years (*p* < 0.05). **(A–D)** Represents the diversity indices of soil fungi and bacteria (Shannon index and Chao1 index), respectively. CK, control; Y, year; 0–10, in soil layer 0–10 cm; 10–20, in soil layer 10–20 cm. Different letters in the same column indicate that the same index has significant differences at the 0.05 level.

Soil microbial diversity of bacteria and fungi changed significantly with increasing years of tillage (Figure 1). Soil α-diversity index of fungal was lower than bacterial. The Shannon and Chao1 indices for both fungi and bacteria increased compared to the control, reaching significant levels at 27 years (*p* < 0.05). The second (10–20 cm) soil index was slightly higher than the first (0–10 cm).

**FIGURE 2:**
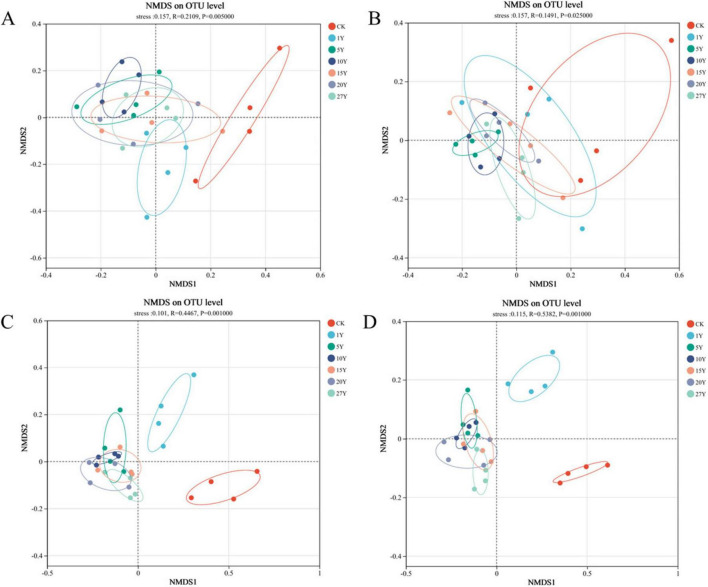
Non-metric multidimensional scaling (NMDS) analysis of soil fungal and bacterial beta diversity in various tillage years. **(A)** 0–10 cm fungal NMDS. **(B)** 10–20 cm fungal NMDS. **(C)** 0–10 cm bacterial NMDS. **(D)** 10–20 cm bacterial NMDS. CK, control; Y, year.

To further explore differences in β-diversity, NMDS analysis was performed. In both fungi and bacteria, Bray-Curtis differences between treatments were separated along the x- or y-axis for various tillage years (Figure 2, stress = 0.157, stress = 0.157, stress = 0.101, stress = 0.115). Furthermore, the ANOSIM highlighted that the soil fungal and bacterial communities with the rice planting (1Y, 5Y, 10Y, 15Y, 20Y, 27Y) were substantially different from those of control site (*p* < 0.05).

### 3.3 Soil microbial community composition

**FIGURE 3:**
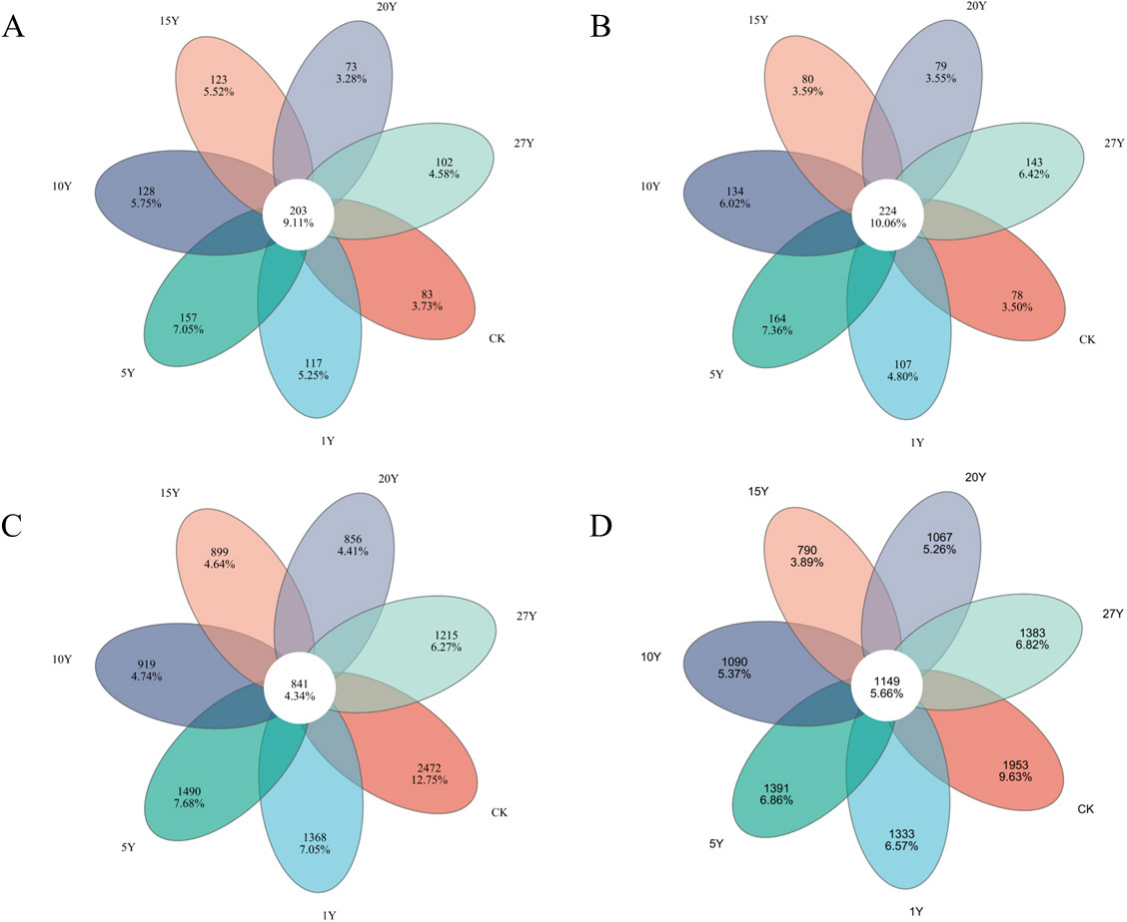
Venn diagram of the soil fungal and bacterial operational taxonomic unit (OTU) in various tillage years. The numbers within circles represent the specific OTU in that treatment, the core number represents the common OTU present in all treatment. **(A)** 0–10 cm fungal. **(B)** 10–20 cm fungal. **(C)** 0–10 cm bacterial. **(D)** 10–20 cm bacterial. CK, control; Y, year.

The Venn diagrams illustrate the abundance of specific bacterial and fungal species (represented as operational taxonomic units, OTUs) associated with different cropping years (Figure 3). In our study, a total of 986 (0–10 cm) and 1,009 (10–20 cm) soil fungal operational taxonomic unit (OTU) species were detected, along with 10,060 (0–10 cm) and 10,156 (10–20 cm) soil bacterial species. Among fungi, core symbionts dominated the study area, accounting for 56.4%–73.6% (0–10 cm) and 57.7%–74.2% (10–20 cm). In contrast, core symbiotic bacterial species accounted for 25.4%–49.6% (0–10 cm) and 37.0%–59.3% (10–20 cm), with neither core symbiotic group being dominant. Across various tillage years within each treatment, detected fungal species exhibited greater treatment specificity than bacterial species.

**FIGURE 4:**
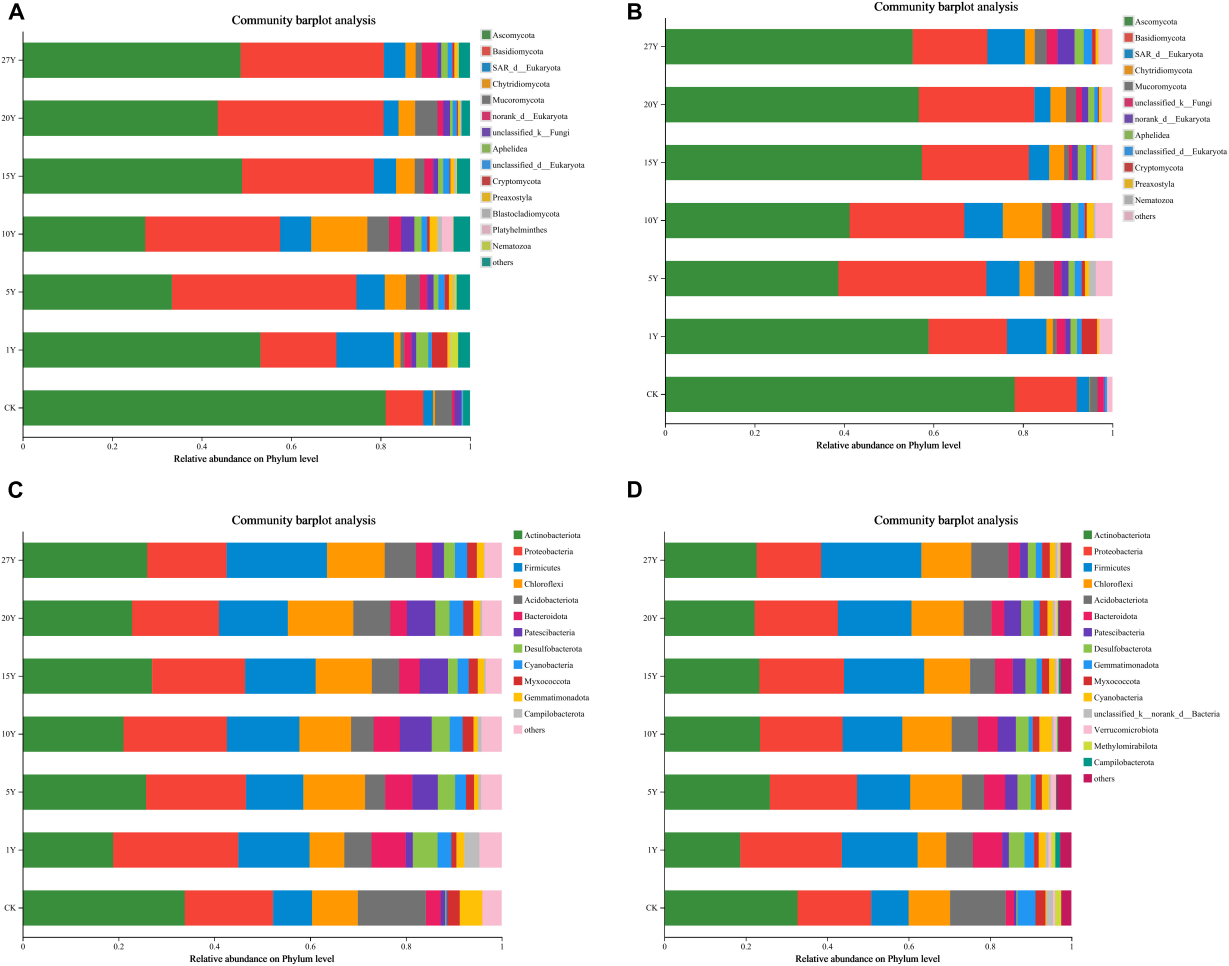
Soil microbial community proportion at phylum level in various tillage years (mean). **(A)** 0–10 cm fungal. **(B)** 10–20 cm fungal. **(C)** 0–10 cm bacterial. **(D)** 10–20 cm bacterial. CK, control; Y, year.

Various tillage years influenced the relative abundance of common fungal and bacterial phylum-level taxa (top 10%). Changes in soil fungal communities across treatments were less pronounced than those in bacterial communities. Specifically, within fungal communities: In the 0–10 cm soil layer, the Ascomycota phylum (27.40%–53.16%) was relatively dominant (Figure 4), followed by Basidiomycota (16.98%–41.27%) and the SAR superphylum (3.35%–12.85%). In the 10–20 cm soil layer, the Ascomycota phylum (38.74%–58.92%) was dominant, followed by Basidiomycota (16.67%–33.11%) and the SAR superphylum (3.59%–8.81%). In contrast, bacterial community diversity showed an increasing trend across different tillage years. In the 0–10 cm soil layer, the most abundant bacterial phyla were: Actinobacteria (18.89%–27.00%), Proteobacteria (16.57%–26.13%), Firmicutes (11.97%–20.95%), and Chloroflexi (7.26%–13.65%) (Figure 4). In the 10–20 cm soil layer, bacterial phyla were dominated by Actinobacteria (18.64%–25.92%), Proteobacteria (15.85%–24.97%), Firmicutes (13.04%–24.60%), and Chloroflexi (7.04%–12.79%).

### 3.4 Influence mechanism of various tillage years on soil microbial community diversity

**FIGURE 5:**
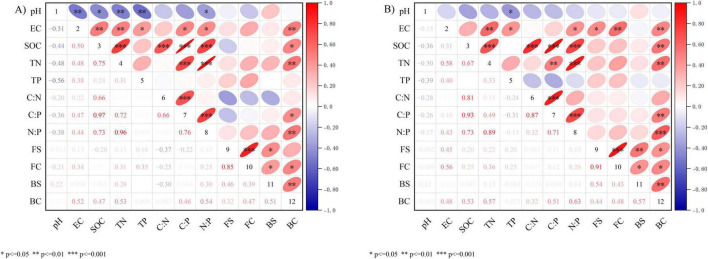
Heat map of correlation between soil physical and chemical properties and alpha diversity. **(A)** 0–10 cm. **(B)** 10–20 cm. pH, soil pH value; EC, soil electrical conductivity; SOC, soil organic carbon content; TN, soil total nitrogen content; TP, soil total phosphorus content; C:N, soil C:N ratio; C:P, soil C:P ratio; N:P, soil N:P ratio.

The correlation between microbial diversity and key physicochemical properties of soil communities is shown in Figure 5. Analysis results for both soil layers were largely consistent: pH showed significant negative correlations with EC, SOC, TN, TP, and other parameters (*p* < 0.05, *p* < 0.01); EC showed significant positive correlations with SOC, TN, TP, C:P, N:P, and the bacterial Chao1 (BC) index (*p* < 0.05, *p* < 0.01); SOC exhibited extremely significant positive correlations with TN, C:N, C:P, and N:P (*p* < 0.001), and a significant positive correlation with BC (*p* < 0.05, *p* < 0.01). The fungal Chao1 (FC) index showed a significant positive correlation with EC (*p* < 0.01), while BC exhibited significant positive correlations with EC, SOC, TN, C:P, N:P, FC, and BS (*p* < 0.05, *p* < 0.01, *p* < 0.001).

**FIGURE 6:**
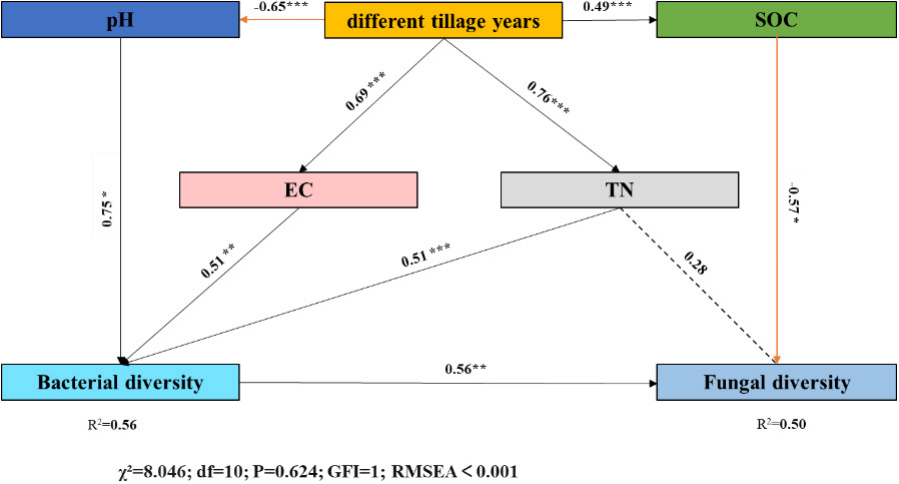
Structural equation modeling was employed to elucidate the direct and indirect effects of long-term tillage on soil fungal and bacterial diversity. The values on arrows represent standardized path coefficients, with asterisks denoting significant relationships (***p < 0.001, **p < 0.01, *p < 0.05). The R^2^ values within rectangles indicate the proportion of variance explained in relationships with other variables. Gray, orange, and dashed arrows denote significantly positive, significantly negative, and non-significant effects at the 0.05 level, respectively. Line thickness reflects effect strength. The χ^2^ value and *P*-value serve as model fit statistics (χ^2^ = 8.046, df = 10, *p* = 0.624). The chi-square test (with a *P*-value range of 0.05 < *p* ≤ 1.00 indicating good model fit) demonstrated that the structural equation model fits the data well.

The final SEM model revealed both direct and indirect effects of long-term rice cultivation on fungal and bacterial diversity: soil total nitrogen, pH, and electrical conductivity collectively explained 56% of bacterial variance (Figure 6). Cultivation duration exerted significant positive indirect effects on soil bacterial diversity (0.75 mediated by soil pH; 0.51 mediated by soil EC; 0.51 mediated by soil total nitrogen). Results indicate that long-term cultivation-induced decreases in pH and increases in electrical conductivity and soil total nitrogen trigger enhanced bacterial diversity. Cultivation duration exerted a significant negative indirect effect (−0.57, mediated by soil organic carbon) and a significant positive indirect effect (0.28, mediated by soil total nitrogen) on soil fungal diversity. These findings suggest that long-term cultivation induces increased fungal and bacterial diversity.

## 4 Discussion

### 4.1 Effect of various tillage years on soil microbial diversity

Long-term rice cropping affects sandy paddy soil through manual tillage practices and the combined application of farmyard manure and chemical fertilizers and irrigation. The soil microenvironment undergoes changes due to the gradual accumulation of salts from chemical and organic fertilizers, coupled with the increasing accumulation of root residues and plant debris over the years. Irrigation practices also influence soil microbial communities, particularly in arid regions where irrigation can improve soil moisture conditions, thereby promoting microbial growth. Such alterations affect soil carbon and nitrogen content as well as pH levels, subsequently altering the diversity and community composition of soil bacteria and fungi.

In many ecosystems, bacterial α-diversity exceeds that of fungi (Anderson et al., 2017; Chen et al., 2020). This disparity also persists in sandy agricultural ecosystems, a finding confirmed by this study. This occurs because fungi typically exhibit lower nutritional requirements than bacteria (Zhou et al., 2017), whereas bacteria grow more rapidly and prefer substrates with low carbon-to-nitrogen ratios. In this study, both fungal and bacterial α-diversity initially showed an increasing trend, primarily due to the low levels of various indicators in sandy soils. Subsequent continuous tillage and fertilization led to increases in these indicator levels. Research indicates (Dong et al., 2017) that as the duration of continuous cropping increases, fungal diversity in cultivated soil tends to rise with the medicinal plant American ginseng. Based on long-term field trials (Yu et al., 2025), it was found that chemical fertilizer application had no significant effect on soil bacterial diversity, whereas organic fertilizer alone significantly enhanced soil bacterial diversity.

A global meta-analysis indicates (Li et al., 2020) that no-till straw incorporation significantly impacts soil microbial diversity. A meta-analysis of Chinese farmlands reveals (Wang Y. Z. et al., 2021) that no-till significantly increases the fungal Shannon index, while straw incorporation markedly elevates both bacterial Shannon and Simpson indices. Litter accumulation promotes soil organic carbon formation and influences soil microbial communities (Wang L. Y. et al., 2021). In this study, rice straw remaining after harvest was either baled for self-use or sold. The rice stubble was left in the field until the following year when it was plowed under, forming litter that, together with fertilization, influenced soil microbial α-diversity.

### 4.2 Effects of various tillage years on soil microbial community composition

The microbial OTU taxonomic composition revealed distinct differences in specificity along environmental gradients. Venn diagrams and community composition charts indicate that soil bacteria exhibit lower specificity and greater adaptability than fungal species, whereas soil fungal species demonstrate greater distinctiveness and specificity at regional scales. Fungi were dominated by the Ascomycota and Basidiomycota phyla, while bacteria were characterized by the relative dominance of Actinobacteria, Proteobacteria, and Firmicutes. Proteobacteria within the bacterial community play crucial biological roles in soil remediation, organic matter decomposition, and nitrogen cycling, making them key members of the soil microbial community. Soil actinomycetes participate in organic matter decomposition, promoting nutrient release. They also contribute to soil aggregation, enhancing stability and water retention, while their metabolites stimulate plant growth and development (Chu et al., 2007). Among soil fungi, the Basidiomycota and Ascomycota phyla are the primary pathogens causing plant diseases (Xu and Hu, 2021).

In this study, compared with the control, tillage increased the relative abundance of Basidiomycota, Actinobacteria, Firmicutes, and Chloroflexi, while decreasing that of Ascomycota and Proteobacteria. Fertilization, flooding, and litter addition enhanced microbial activity, diversity, and function in sandy soil, promoting the development of beneficial microbial communities. As nutrient enrichment may alter interactions among microbial species, sustained increases in nutrient supply could shift relationships from symbiosis to competition (Hoek et al., 2016), leading to diverse changes in soil microbial communities. Changes in soil fungal and bacterial diversity and dominant communities did not respond uniformly to increasing years of tillage, possibly due to the combined effects of regional, climatic, soil properties, and agricultural management practices.

### 4.3 Relationship of environmental factor with soil microbial community with various tillage years

In paddy agricultural ecosystems, fertilization is a common practice to enhance crop yields. Extensive research has demonstrated that appropriate agricultural management practices positively influence soil nutrients and microorganisms. For instance, applying organic fertilizers and tillage increase soil microbial diversity and organic matter content (Xu et al., 2021; Song et al., 2022). Sustained inputs of organic or inorganic nutrients alter the original ecological succession processes of microorganisms and modify the soil’s physicochemical structure.

Fertilization introduces exogenous organic or inorganic nutrients, altering the original physiological habitat of microorganisms. Proper agricultural management practices can promote microbial growth. The primary indicators driving soil microbial communities vary across ecosystems. For instance, in desert grasslands, soil C:N ratio, salinity, and air temperature are the most critical indicators (Wang S. et al., 2021); whereas in the Namib Desert, plantation forests, dryland soils, and global agricultural ecosystems, pH is the most crucial indicator (Maestre et al., 2015; Zhou et al., 2017; Scola et al., 2018; Dang et al., 2022).

## 5 Conclusion

Continuous rice cultivation in sandy areas affected the diversity and composition of soil microorganisms in sandy farmland ecosystems. Soil bacterial communities were more responsive than fungal communities, reflecting different adaptive strategies to cope with nutrient availability in sandy agroecosystems. Soil pH, EC, SOC, and TN are indicators that influence the composition of soil microbial communities. Tillage fertilization indirectly affected soil bacterial composition by decreasing pH and soil fungal composition by increasing total soil nitrogen. Furthermore, soil bacterial and fungal communities responded differently, bacterial communities showing higher alpha diversity than fungi, and the beta diversity of soil fungi higher than bacteria. In particular, soil bacteria were dominated by the phylum Actinobacteria, while fungi were dominated only by the phylum Ascomycota.

The present study suggests that film-lined rice cultivation should be implemented within a controlled range to maintain soil microbial community composition and improve soil properties in sandy agro-ecosystems. Our study provides important insights into the impacts of film-lined rice cultivation and provides a basis for maintaining soil microbial diversity and sustainable land development in sandy agroecosystems. Based on our findings, the incorporation of crop residues and the application of organic-inorganic fertilizer mixtures over extended periods alter the structure of soil microbial communities. These changes enhance soil fertility and crop yields, providing scientific support for the improved development of plastic-film-covered rice cultivation.

## Data Availability

The original contributions presented in the study are publicly available. This data can be found here: https://www.ncbi.nlm.nih.gov/bioproject/PRJNA1439423.

## Footnotes

1
https://cloud.majorbio.com

2
http://huttenhower.sph.harvard.edu/LEfSe
